# Subjective social position and cognitive function in a longitudinal cohort of older, rural South African adults, 2014–2019

**DOI:** 10.1136/jech-2021-217059

**Published:** 2021-09-23

**Authors:** Lindsay C Kobayashi, Emily P Morris, Guy Harling, Meagan T Farrell, Mohammed U Kabeto, Ryan G Wagner, Lisa F Berkman

**Affiliations:** 1 Department of Epidemiology, University of Michigan, Ann Arbor, Michigan, USA; 2 Department of Psychology, University of Michigan, Ann Arbor, Michigan, USA; 3 Institute for Global Health Research, University College London, London, UK; 4 Harvard Center for Population and Development Studies, Harvard University T H Chan School of Public Health, Cambridge, Massachusetts, USA; 5 MRC/Wits Rural Public Health & Health Transitions Research Unit (Agincourt), University of the Witwatersrand, Johannesburg-Braamfontein, Gauteng, South Africa; 6 Africa Health Research Institute, Durban, KwaZulu-Natal, South Africa; 7 Department of Internal Medicine, University of Michigan Medical School, Ann Arbor, Michigan, USA

## Abstract

**Background:**

The relationship between subjective social position (SSP) and cognitive ageing unclear, especially in low-income settings. We aimed to investigate the relationship between SSP and cognitive function over time among older adults in rural South Africa.

**Methods:**

Data were from 3771 adults aged ≥40 in the population-representative ‘Health and Ageing in Africa: A Longitudinal Study of an INDEPTH Community in South Africa’ from 2014/2015 (baseline) to 2018/2019 (follow-up). SSP was assessed at baseline with the 10-rung MacArthur Network social position ladder. Outcomes were composite orientation and episodic memory scores at baseline and follow-up (range: 0–24). Mortality- and attrition-weighted linear regression estimated the associations between baseline SSP with cognitive scores at each of the baseline and follow-up. Models were adjusted for age, age^2^, sex, country of birth, father’s occupation, education, employment, household assets, literacy, marital status and health-related covariates.

**Results:**

SSP responses ranged from 0 (bottom ladder rung/lowest social position) to 10 (top ladder rung/highest social position), with a mean of 6.6 (SD: 2.3). SSP was positively associated with baseline cognitive score (adjusted β=0.198 points per ladder rung increase; 95% CI 0.145 to 0.253) and follow-up cognitive score (adjusted β=0.078 points per ladder rung increase; 95% CI 0.021 to 0.136).

**Conclusion:**

Independent of objective socioeconomic position measures, SSP is associated with orientation and episodic memory scores over two time points approximately 3 years apart among older rural South Africans. Future research is needed to establish the causality of the observed relationships, whether they persist over longer follow-up periods and their consistency in other populations.

## INTRODUCTION

By 2050, approximately 75% of global dementia cases are expected to occur in low-income and middle-income countries, although socioeconomic influences on cognitive ageing in these settings are understudied.^1^ Traditional socioeconomic position (SEP) indicators, including education, income, occupation and wealth are consistently associated with cognitive ageing outcomes.^2–5^ SEP is thought to affect cognitive ageing through influencing access to health-promoting resources across the life course such as nutrition, housing, social connections, leisure time activities and healthcare.^6–8^ The psychological dimensions of SEP, such as subjective social position (SSP), have been rarely investigated in relation to cognitive ageing.^9 10^ SSP represents an individual’s perception of their relative position within their community or society.^11 12^ SSP is thought to affect health through representing the factors that individuals consider to influence their social position, which may not be fully captured by traditional SEP indicators, and through psychosocial stress that may be induced through the experience of perceiving a low social position.^11–14^

SSP has been associated with a range of health outcomes among older adults, mostly in high-income settings, including depression, hypertension, diabetes, elevated cortisol levels and functional mobility decline.^15–19^ Several of these outcomes are risk factors for cognitive decline, impairment and dementia.^20^ To the best of our knowledge, only two studies have investigated the associations between SSP and cognitive ageing.^9 10^ The first found that SSP in relation to ‘society’ in the USA was strongly associated with memory function at baseline, but not with memory change over a 6-year follow-up in a population-representative sample of 8530 adults aged ≥65.^9^ The second found that low SSP relative to one’s local community, but not all of Hong Kong, was strongly associated with accelerated cognitive decline over a 4-year follow-up among adults aged ≥65 in Hong Kong, China.^10^

In this study, we focus on South Africa, which has a history of legislated racial segregation and discrimination due to apartheid and its precursors, and racialised socioeconomic inequality that has persisted since the end of apartheid in 1994.^21–23^ The Black population was excluded from the education system during apartheid, with ‘Bantu’ schools providing a basic level of skills designed to keep Black South Africans in low-paying manual labour positions.^24^ Unemployment was and remains high in South Africa, with sources of employment and income continuing to be volatile in rural regions where cyclical and migratory work such as in mining, farming and domestic labour are common.^25–27^ The associations between SSP and cognitive ageing outcomes among older, Black South Africans who lived through apartheid are unknown.

We aimed to investigate the associations between SSP within one’s village and cognitive function over two time points in a longitudinal study of men and women aged ≥40 living in the rural Agincourt subdistrict of Mpumalanga province, South Africa, from 2014/2015 to 2018/2019. We hypothesised that higher SSP would be associated with higher baseline and follow-up cognitive function, independent of objective SEP measures and other confounders.

## METHODS

### Study design and sample

**Figure 1:**
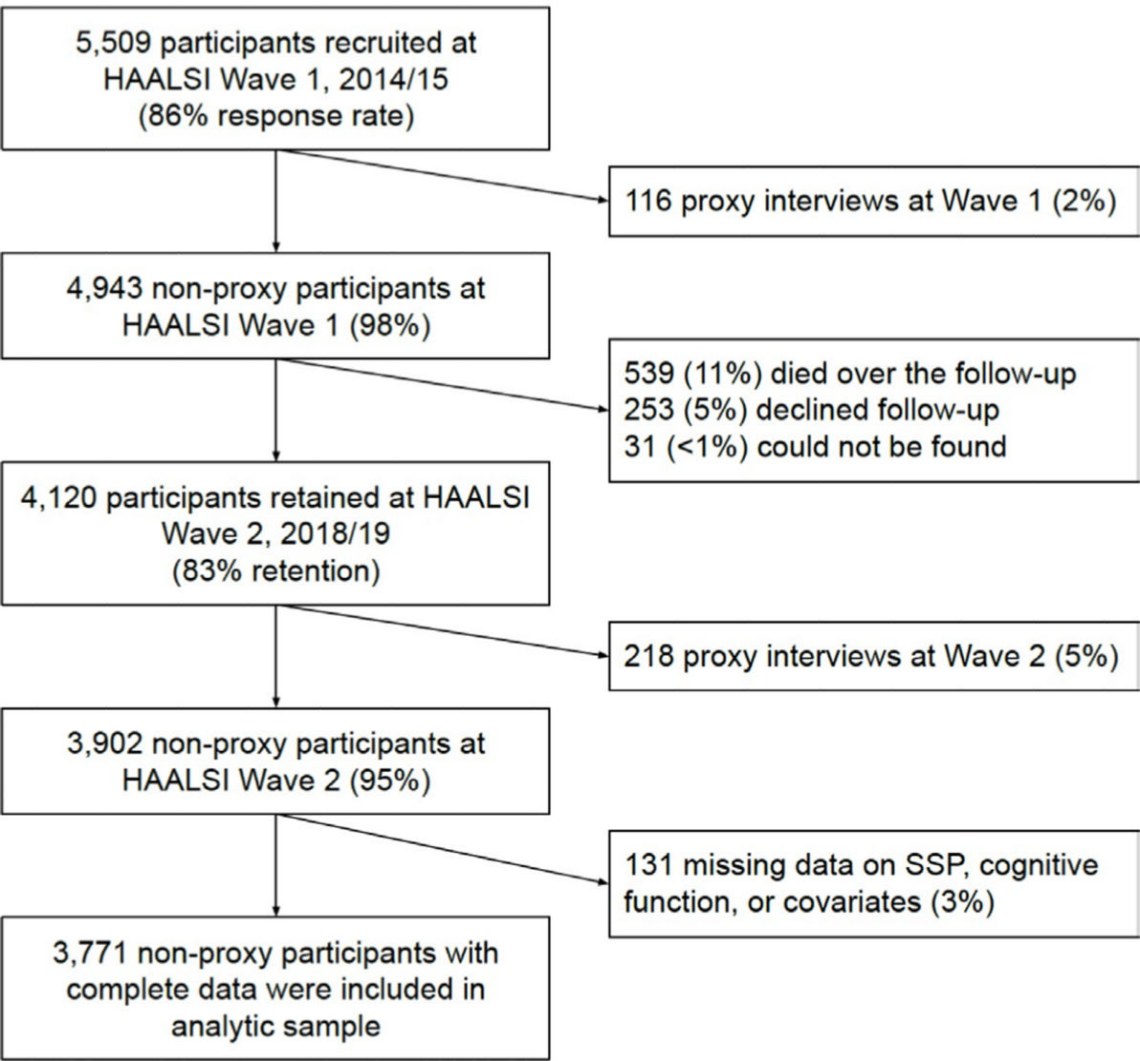
Study flow diagram. Among the included sample, mean follow-up time was 3.67 years (SD: 0.24 years; range: 2.95–4.82 years). HAALSI, Health and Ageing in Africa: A Longitudinal Study of an INDEPTH Community in South Africa; SSP, subjective social position.

Data were from in-person interviews with adults aged ≥40 in ‘Health and Ageing in Africa: A Longitudinal Study of an INDEPTH Community in South Africa’ (HAALSI, response rate=86%).^28^ Baseline interviews were conducted from November 2014 to November 2015, and follow-up interviews from October 2018 to November 2019. The lower limit of the eligible age range reflects the life expectancy of South Africans, at 60.6 years for men and 64.3 years for women in 2015.^29^ HAALSI represents of a population of ~1 16 000 people living in 31 villages in the Agincourt subdistrict, Mpumalanga province, South Africa.^28 30^ Agincourt is part of a former ‘homeland’ region, where Black South Africans were forcibly moved to during apartheid on the basis of their racial and ethnic identity.^30^ During apartheid, opportunities for social mobility were severely limited through restricted access to education and occupational opportunities.^21^ Since the end of apartheid in 1994, social and economic conditions in Agincourt have improved, but there remain gaps in basic services such as piped water and tarred roads.^28 30^ Approximately one-third of the Agincourt population are former refugees due to the civil war in neighbouring Mozambique from 1977 to 1992. These individuals generally had worse access to health and social opportunities than their South African-born counterparts. ^31^ A total of 3771 participants were included in the final sample (figure 1).

### Exposure: SSP

SSP was assessed using the 10-rung MacArthur Network social position ladder, using the participant’s village as the reference frame.^13 18^ Participants were shown an image of a 10-rung ladder, asked to imagine that it represents where people stand in their village, and to indicate where on the ladder they felt they stood relative to others in their village. The ladder measure generated a continuous SSP variable with a range from 0 (bottom rung) to 10 (top rung).

### Outcomes: cognitive function at the baseline and follow-up

Cognitive function was assessed at baseline (2014/15) and follow-up (2018/19) using measures adapted from the US Health and Retirement Study (HRS)^31^: orientation (ability to state the current day, month, year and South African president; four points total) and episodic memory (immediate and delayed recall of a 10-word list read out loud by the interviewer; 20 points total; delays were 1 min at baseline and 6 min at follow-up). These measures are used in HRS International Partner Studies around the world^32^ and were selected as the inability to orient oneself to time and place and loss in memory function are hallmark signs of dementia, including Alzheimer’s disease, and are highly sensitive to ageing-related change. ^33^ Psychometric data on the HAALSI cognitive battery are available elsewhere.^34^

### Covariates

Potential confounders of the SSP–cognitive health relationship were measured in the baseline interview. Demographic covariates were: age (continuous); age^2^ (continuous, to account for non-linear effects of age on cognition); sex (male; female); country of birth (South Africa; Mozambique or other). Socioeconomic and social covariates were: skill level of father’s main job during childhood as a proxy for childhood socioeconomic conditions^34^ (skill levels one through four of the International Standard Classification of Occupations (ISCO) 2008; other; don’t know); education (no formal education; some primary (1–7 years); some secondary (8–11 years); secondary or more (12+years)); self-reported literacy (cannot read and/or write; can read and write); current employment (employed full time or part time; retired; homemaker; unemployed); marital status (married or living as married; never married; separated/deserted; divorced; widowed); asset-based household wealth quintile. Please see online supplemental methods 1 for additional details on the ISCO 2008. Pre-existing health conditions and behaviours included: self-rated health today compared with 1 year ago (much worse; worse; same; better; much better), frequency of alcohol intake (less than five vs five or more drinks/week), presence of depressive symptoms according to a modified 7-item Center for Epidemiologic Depression scale (yes; no), hypertension (yes; no) and diabetes (yes; no). Hypertension was defined as a measured mean systolic blood pressure >140 mm Hg or mean diastolic blood pressure >90 mm Hg, or controlled blood pressure with self-reported use of hypertensive medication. Diabetes was defined as any of a fasting glucose of ≥7.0 mmol/L, a random plasma glucose ≥11.1 mmol/L, a self-reported physician diagnosis of diabetes, or self-reported use of a prescribed diabetes medication.

### Statistical analyses

Linear regression was used to estimate the relationships between SSP and cognitive function score at the baseline and the follow-up. Models for both outcomes were iteratively adjusted for (1) demographic covariates (model set 1); (2) demographic plus socioeconomic and social covariates (model set 2) and (3) demographic, socioeconomic and social, and health-related covariate (model Set 3). Because the goal of model adjustment was to control for confounding, we did not adjust the models predicting follow-up cognitive score for the baseline cognitive score, as baseline cognitive function may be a mediator of any association between baseline SSP and cognitive function at the follow-up.^35 36^ All models incorporated inverse probability weights that accounted for the probabilities of mortality and attrition over the follow-up.^37^ Please see online supplemental methods 2 and online supplemental tables 1 and 2 for a description of the methods used to create the weights. We conducted a sensitivity analysis with the models predicting follow-up cognitive function score adjusted for the baseline cognitive function score, to evaluate the association of baseline SSP with follow-up cognitive function, net of baseline cognitive function. All analyses were conducted using Stata V.17.0SE.

## RESULTS

Characteristics of the sample and mean SSP scores according to sample characteristics are shown in table 1. SSP responses ranged from 0 (bottom rung of ladder) to 10 (top rung of ladder), with a mean of 6.6 (SD: 2.3), median of 7, and IQR from 5 to 8. Characteristics of the HAALSI sample compared with the national South African population aged ≥40 are shown in online supplemental table 3. Cognitive scores were normally distributed at both time points, with a baseline median (IQR) of 12 (9–14) and a follow-up median (IQR) of 13 (10–16).

SSP was positively associated with baseline cognitive function, with an average 0.300-point increase in score (95% CI: 0.244 to 0.356) per one-rung SSP ladder position increase after adjusting for age, age^2^, sex and country of birth in model 1 (table 2). After adjusting for socioeconomic and social factors in model 2, this estimate was attenuated by approximately one-third to 0.210 (95% CI 0.156 to 0.264), while additional adjustment for health-related factors negligibly affected the final estimate in Model 3 (0.198; 95% CI 0.144 to 0.253; table 2). Full model estimates are available in online supplemental table 4.

SSP was also positively associated with follow-up cognitive function, but with a weaker magnitude of association than with baseline cognitive function, with an average 0.168-point increase in score (95% CI 0.110 to 0.226) per one-rung SSP ladder position increase in model 1 (table 3). This estimate was attenuated by approximately half after adjustment for socioeconomic, social and health factors in model 3 (0.078; 95% CI 0.021 to 0.136; table 3). Full model estimates are available in online supplemental table 5.

When the models shown in table 3 were rerun with adjustment for baseline cognitive function score, as a sensitivity analysis, the estimates were somewhat attenuated and became non-statistically significant for models 2 and 3: model 1: β=0.098 (95% CI 0.200 to 0.268); model 2: β=0.049 (95% CI −0.006 to 0.105); Model 3: β=0.048 (95% CI −0.009 to 0.104). The Pearson’s correlation coefficient for the baseline and follow-up cognitive function scores was 0.39 (p<0.0001).

## DISCUSSION

In this population-representative cohort of older adults in a low income, rural region of South Africa, we observed that higher SSP was associated with better episodic memory and orientation function at baseline and at a 3-year follow-up, independent of covariates including traditional objective SEP measures. Objective SEP measures, but not health-related measures, were confounders of the relationships between SSP and cognitive outcomes at both time points. The relationship between SSP and cognitive function was weaker at the follow-up than at the baseline. These findings are among the first to indicate that SSP is associated with cognitive health among older adults in rural South Africa. Future studies in similar settings should consider including SSP as a socioeconomic covariate alongside objective SEP measures, and further investigate the longer-term relationships between SSP and cognitive ageing, including risks of cognitive impairment and dementia.

### Comparison with existing literature and potential explanations

Our results indicating that SSP was positively associated with baseline cognitive function is consistent with the prior study of SSP and cognitive ageing in the US HRS.^9^ Our findings are also consistent with the study of older adults in Hong Kong for the community-level but not society-level SSP reference frame.^10^ In contrast to the US study, our study and the Hong Kong study represented low-income older populations with histories of socioeconomic deprivation. It is possible that SSP is influenced by different factors across older populations from different socioeconomic and cultural contexts. Additionally, the SSP reference frame may influence results. Results from these studies indicate that local reference frames are associated with cognitive outcomes (eg, one’s village in rural South Africa, one’s self-defined community in Hong Kong), while larger societal reference frames are not (eg, all of Hong Kong, US society). Further research is needed to better understand how older adults from diverse country contexts conceptualise their SSP, and how the relationships between SSP and SEP may differ across populations.

The potentially unmeasured aspects of SEP that are captured by SSP in low-income settings may reflect cognitive reserve. Cognitive reserve is a theoretical construct that is thought to reflect the brain’s ability to maintain cognitive function despite the presence of neurodegenerative pathology.^38^ Cognitive reserve is strongly associated with objective SEP measures, which are thought to contribute to cognitive reserve through providing access to cognitively stimulating social and leisure-time activities, healthcare, nutrition and other cognitively protective resources.^38^ As such, conditional on objective SEP, high SSP may reflect cognitive reserve obtained through socioeconomic or social means that are not captured by SEP measures. Future research should explicitly examine how SSP is associated with cognitive reserve markers, particularly in low-income settings that will experience a heavy future burden of ageing-related cognitive decline.

A final potential explanation for our findings is that low SSP may induce greater social stress in low-income settings than in high-income settings, possibly due to the lower absolute socioeconomic welfare experienced by those in low-income settings. Future research should interrogate the construct of SSP in low-income and middle-income settings, and the associations of SSP with psychosocial stress pathways that are thought to lead to several chronic diseases, including dementia.^20 39 40^ Cross-national comparative studies of the associations of SSP with health outcomes, net of contextually relevant SEP measures, may also be informative for evidence triangulation on the potential role of SSP in health outcomes.

### Limitations and strengths

This study is limited to the cognitive domains of orientation and episodic memory. SSP may be differentially associated with other cognitive function domains, such as executive function and processing speed. However, the domains we studied are highly sensitive to ageing-related change, and impairments in orientation and episodic memory are hallmark signs of Alzheimer’s disease and related dementias.^33^ As we only had two time points of outcome measurement, we were unable to study rate of change over time in cognitive function using standard methods to account for multiple within-person outcome measures over time.^37^ We were unable to study incident cognitive impairment or dementia as outcomes, as a longer follow-up time is needed allow enough cases to occur for sufficient statistical power. There was a longer delay between the immediate and delayed recall trials at the follow-up then at the baseline, which was more consistent with the HRS delay of approximately 5 min. The longer delay may have made the delayed recall trial more difficult at the follow-up, but the potential impact on our estimated measures of association is difficult to evaluate. Our cognitive scores may be sensitive to measurement error, which we expect would be random with respect to SSP and, if present, would most likely have resulted in our results underestimating the true magnitudes of associations. Our analytic sample was restricted to non-proxy respondents, excluding the most cognitively impaired individuals. If there truly is a positive relationship between SSP and cognitive function, exclusion of these individuals would further bias our results towards the null.

Our data were observational, and sources of bias such as unmeasured confounding and study attrition are threats to causal inference. We addressed these limitations as best possible through model adjustments and weighting to account for mortality and attrition. One concern is that unmeasured cognitive function prior to the study baseline is a source of confounding. If this is the case, then our results may reflect reverse causation. To explore this possibility in a sensitivity analysis, we adjusted for baseline cognitive function in the model predicting cognitive function at the follow-up, as the baseline scores would be correlated with unmeasured values of cognitive function prior to the baseline. In this analysis, the estimate for SSP was somewhat attenuated and non-statistically significant. However, it is not possible to ascertain whether this attenuation was due to improved confounder control or because adjustment for baseline cognitive function removed some of the total effect of SSP on cognitive function at the follow-up. Future research should establish the temporality of relationships between SSP and cognitive outcomes using additional repeated measures.

Strengths include our use of a widely used, validated and reliable measure of SSP.^11^ We had longitudinal data on a large, population-representative sample, including rich data on covariates and cognitive function that are harmonised with the US HRS and its International Partner Studies.^32^ This study improves global representation in the body of social epidemiological evidence on cognitive ageing and is the first that we are aware of to identify an association between SSP and cognitive outcomes in a low-income, rural, southern African setting. The social and economic circumstances of this study population are broadly generalisable to other rural regions of South Africa at similar levels of economic development. Findings of this study should be valuable for hypothesis generation to better understand SSP as a meaningful measure of social position in older, low-income populations and its potential relationships with health outcomes.

## Conclusion

SSP was associated with orientation and episodic memory scores over time among low-income older adults in a rural region of South Africa. We observed strong associations between SSP and cognitive function, which attenuated over time but persisted after adjustment for objective SEP measures. Future research is needed to establish the causality of the observed relationships, whether they persist over longer follow-up periods, their consistency in other populations, and whether they extend to other cognitive domains and outcomes.

## Supplementary Material

Supplementary Material

## Figures and Tables

**Table 1:** Characteristics of the sample, HAALSI, Agincourt subdistrict, Mpumalanga, South Africa, 2014–2019

Characteristic	N (%)	Mean (SD) SSP
	
	3771 (100%)	6.6 (2.3)

Demographic characteristics		
Age		
Mean (SD)	60.1 (12.2)	−0.10*
P value		<0.0001
Sex		
Male	1657 (43)	6.66 (2.35)
Female	2114 (56)	6.53 (2.19)
P value	-	0.08
Country of birth		
South Africa	2648 (70)	6.83 (2.24)
Mozambique or other	1123 (30)	6.01 (2.20)
P value	-	<0.0001
Socioeconomic and social characteristics		
Skill level of father’s main job during childhood		
Skill level 1 (unskilled manual labour)	1095 (29)	6.63 (2.22)
Skill level 2 (mining or service sector work)	1677 (45)	6.57 (2.24)
Skill level 3 (traditional healers and small business assistants)	113 (3)	6.69 (2.13)
Skill level 4 (professional or managerial work)	108 (3)	6.29 (2.25)
Other	408 (11)	7.04 (2.43)
Don’t know	370 (10)	6.07 (2.25)
P value		<0.0001
Education		
No formal education	1605 (43)	6.20 (2.24)
Some primary (1–7 years)	1343 (36)	6.65 (2.20)
Some secondary (8–11 years)	464 (12)	6.88 (2.30)
Secondary or more (≥12 years)	359 (10)	7.66 (2.11)
P value		<0.0001
Self-reported literacy		
Can read and write	2061 (55)	6.89 (2.23)
Cannot read and/or write	1710 (45)	6.21 (2.25)
P value		<0.0001
Marital status		
Married or living as married	2024 (54)	6.79 (2.27)
Never married	181 (5)	6.70 (2.43)
Separated/deserted	319 (8)	6.09 (2.15)
Divorced	158 (4)	6.50 (2.23)
Widowed	1089 (29)	6.33 (2.21)
P value		<0.0001
Employment status		
Employed part or full-time	657 (17)	6.87 (2.24)
Not working	2695 (71)	6.50 (2.28)
Homemaker	419 (11)	6.67 (2.11)
P value		0.0004
Household asset quintile		
1 (poorest)	732 (19)	5.84 (2.30)
2	735 (19)	6.33 (2.23)
3	740 (20)	6.63 (2.23)
4	781 (21)	6.88 (2.19)
5 (richest)	783 (21)	7.18 (2.15)
P value		<0.0001
Health-related characteristics		
Self-rated health today, compared with year ago		
Much worse	95 (3)	6.58 (2.70)
Worse	546 (14)	6.17 (2.19)
Same	2290 (61)	6.70 (2.25)
Better	612 (16)	6.34 (2.24)
Much better	228 (6)	7.07 (2.24)
P value		<0.0001
Alcohol intake frequency		
<5 days per week	3553 (94)	6.61 (2.27)
≥5 days per week	218 (6)	6.13 (2.14)
P value		0.002
Presence of depressive symptoms		
Yes	619 (16)	5.78 (2.19)
No	3152 (84)	6.74 (2.24)
P value		<0.0001
Diabetes		
Yes	382 (10)	6.54 (2.18)
No	3131 (83)	6.58 (2.27)
Unknown	258 (7)	6.74 (2.23)
P value		0.472
Hypertension		
Yes	2347 (62)	6.61 (2.24)
No	1379 (37)	6.55 (2.29)
Unknown	45 (1)	6.51 (2.67)
P value		0.748

P values are estimated from t-tests for binary covariates, one-way ANOVA for categorical covariates and Pearson’s correlation for continuous covariates.

* Estimate shown is a Pearson’s correlation coefficient.

ANOVA, analysis of variance; HAALSI, Health and Ageing in Africa: A Longitudinal Study of an INDEPTH Community in South Africa; SSP, subjective social position.

**Table 2:** Linear regression models predicting baseline cognitive function score, HAALSI, Agincourt subdistrict, Mpumalanga, South Africa, 2014–19, N=3771

		Change in baseline cognitive score per one-rung increase on the SSP ladder	
		
Model	Adjustments	β	95% CI

1	Age, age^2^, sex, country of birth	0.300	0.244 to 0.356
2	Model 1+ socioeconomic and social factors*	0.210	0.156 to 0.264
3	Model 2+ health-related factors†	0.198	0.144 to 0.253

All models incorporate IPWs for mortality and attrition.

* Adjusted for model 1 covariates, plus socioeconomic and social factors (father’s main job during childhood, education, literacy, marital status, employment status, household asset quintile).

† Adjusted for model 1 and 2 covariates, plus health-related factors (self-rated health today compared with 1 year ago, alcohol intake frequency, number of depressive symptoms, diabetes, hypertension).

HAALSI, Health and Ageing in Africa: A Longitudinal Study of an INDEPTH Community in South Africa; IPW, inverse probability weights; SSP, subjective social position.

**Table 3:** Linear regression models predicting follow-up cognitive function score, HAALSI, Agincourt subdistrict, Mpumalanga, South Africa, 2014–2019, N=3771

		Change in follow-up cognitive score per one-rung increase on the SSP ladder	
		
Model	Adjustments	β	95% CI

1	Age, age^2^, sex, country of birth	0.168	0.110 to 0.226
2	Model 1+ socioeconomic and social factors*	0.081	0.024 to 0.138
3	Model 2+ health-related factors†	0.078	0.021 to 0.136

All models incorporate IPWs for mortality and attrition.

* Adjusted for model 1 covariates, plus socioeconomic and social factors (father’s main job during childhood, education, literacy, marital status, employment status, household asset quintile).

† Adjusted for model 1 and 2 covariates, plus health-related factors (self-rated health today compared with 1 year ago, alcohol intake frequency, number of depressive symptoms, diabetes, hypertension).

HAALSI, Health and Ageing in Africa: A Longitudinal Study of an INDEPTH Community in South Africa; IPW, inverse probability weights; SSP, subjective social position.

## What is already known on this subject

- Populations of many low-income settings, like much of rural South Africa, are rapidly ageing, yet little is known about the social factors that influence health during ageing in these settings.
- Research in high-income countries has demonstrated that subjective social position is independently associated with a range of physical and mental health outcomes, including cognitive function.
- The independent relationships between subjective social position and cognitive ageing outcomes in low-income, rural populations are unknown.

## What this study adds

- We newly identified that subjective social position was positively associated with cognitive function over two time points in older adults living in rural South Africa.
- The associations between subjective social position and cognitive outcomes were independent of measured aspects of absolute and relative socioeconomic position.
- Subjective social position may play a role in cognitive ageing among low-income older adults, potentially through capturing aspects of social position not reflected in traditional objective measures and through the psychosocial stress of occupying a low social position.

## References

[1] Prince M, Bryce R, Albanese E, The global prevalence of dementia: a systematic review and meta-analysis. Alzheimers Dement 2013;9:63–75. 23305823 10.1016/j.jalz.2012.11.007

[2] Clouston SAP, Kuh D, Herd P, Benefits of educational attainment on adult fluid cognition: international evidence from three birth cohorts. Int J Epidemiol 2012;41:1729–36. 23108707 10.1093/ije/dys148 PMC3535750

[3] Marden JR, Tchetgen Tchetgen EJ, Kawachi I. Contribution of socioeconomic status at 3 life-course periods to late-life memory function and decline: early and late predictors of dementia risk. In: American Journal of Epidemiology, 2017. 10.1093/aje/kwx155 PMC5859987 28541410

[4] Beydoun MA, Beydoun HA, Gamaldo AA, Epidemiologic studies of modifiable factors associated with cognition and dementia: systematic review and meta-analysis. BMC Public Health : 2014;14:643. 24962204 10.1186/1471-2458-14-643 PMC4099157

[5] Mejia-Arango S, Garcia-Cifuentes E, Samper-Ternent R, Socioeconomic disparities and gender inequalities in dementia: a community-dwelling population study from a middle-income country. J Cross Cult Gerontol 2021;36:105–118. 33247379 10.1007/s10823-020-09418-4

[6] Lövdén M, Fratiglioni L, Glymour MM, Education and cognitive functioning across the life span. Psychol Sci Public Interest 2020;21:6–41. 32772803 10.1177/1529100620920576 PMC7425377

[7] Phelan JC, Link BG, Tehranifar P. Social conditions as fundamental causes of health inequalities: theory, evidence, and policy implications. J Health Soc Behav 2010;51 Suppl:S28–40. 10.1177/0022146510383498 20943581

[8] Deckers K, Cadar D, van Boxtel MPJ, Modifiable risk factors explain socioeconomic inequalities in dementia risk: evidence from a population-based prospective cohort study. J Alzheimers Dis : 2019;71:549–57. 31424404 10.3233/JAD-190541 PMC6839472

[9] Zahodne LB, Kraal AZ, Zaheed A, Subjective social status predicts late-life memory trajectories through both mental and physical health pathways. Gerontology 2018;64:466–74. 29597204 10.1159/000487304

[10] Kim JH, Sumerlin TS, Goggins WB, Does low subjective social status predict cognitive decline in Chinese older adults? A 4-year longitudinal study from Hong Kong. Am J Geriatr Psychiatry 2021. doi:10.1016/j.jagp.2021.01.014. [Epub ahead of print: 23 Jan 2021]. PMC8298609 33563520

[11] Cundiff JM, Smith TW, Uchino BN, Subjective social status: construct validity and associations with psychosocial vulnerability and self-rated health. Int J Behav Med 2013;20:148–58. 22200973 10.1007/s12529-011-9206-1

[12] Wilkinson RG. Health, hierarchy, and social anxiety. In: Annals of the new York Academy of sciences. . New York Academy of Sciences, 1999: 896. 48–63. 10681887 10.1111/j.1749-6632.1999.tb08104.x

[13] Operario D, Adler NE, Williams DR. Subjective social status: reliability and predictive utility for global health. Psychol Health 2004;19:237–46.

[14] Adler NE, Newman K. Socioeconomic disparities in health: pathways and policies. Health Aff 2002;21:60–76. 10.1377/hlthaff.21.2.60 11900187

[15] Euteneuer F. Subjective social status and health. Curr Opin Psychiatry 2014;27:337–43. 25023883 10.1097/YCO.0000000000000083

[16] Miyakawa M, Magnusson Hanson LL, Theorell T, Subjective social status: its determinants and association with health in the Swedish working population (the SLOSH study). Eur J Public Health 2012;22:593–7. 21646364 10.1093/eurpub/ckr064

[17] Weiss D, Weiss M. The interplay of subjective social status and essentialist beliefs about cognitive aging on cortisol reactivity to challenge in older adults. Psychophysiology 2016;53:1256–62. 27159187 10.1111/psyp.12667 PMC4949085

[18] Demakakos P, Nazroo J, Breeze E, Socioeconomic status and health: the role of subjective social status. Soc Sci Med 2008;67:330–40. 18440111 10.1016/j.socscimed.2008.03.038 PMC2547480

[19] Adler N, Singh-Manoux A, Schwartz J, Social status and health: a comparison of British civil servants in Whitehall-II with European-and African-Americans in cardia. Soc Sci Med : 2008;66:1034–45. 18180089 10.1016/j.socscimed.2007.11.031

[20] Livingston G, Huntley J, Sommerlad A, Dementia prevention, intervention, and care: 2020 report of the Lancet Commission. Lancet 2020;396:413–46. 32738937 10.1016/S0140-6736(20)30367-6 PMC7392084

[21] Nations United. Special Committee on the policies of apartheid of the government of the Republic of South Africa. Report of the special Committee on the policies of apartheid of the government of the Republic of South Africa, 1969.

[22] Leibbrandt M, Finn A, Woolard I. Describing and decomposing post-apartheid income inequality in South Africa. Dev South Afr 2012;29:19–34.

[23] Wolpe H. Capitalism and cheap labour-power in South Africa: from segregation to apartheid 1. Econ Soc 1972;1:425–56.

[24] Christie P, Collins C. Bantu education: apartheid ideology or labour reproduction? Comp Educ 1982;18:59–75.

[25] Ranchhod V. Earnings volatility in South Africa. SALDRU Work Pap, 2013. Available: https://ideas.repec.org/p/ldr/wpaper/121.html [Accessed 29 Jul 2021].

[26] Delius P. The History of Migrant Labor in South Africa (1800–2014).. In: Oxford research encyclopedia of African history. Oxford University Press, 2017.

[27] The World Bank. Unemployment, total (% of total labor force) (modeled ILO estimate) -South Africa. Int. Labour Organ. ILOSTAT database, 2020. Available: https://data.worldbank.org/indicator/SL.UEM.TOTL.ZS?locations=ZA [Accessed 29 Jul 2021].

[28] Gómez-Olivé FX, Montana L, Wagner RG, Cohort profile: health and ageing in Africa: a longitudinal study of an indepth community in South Africa (HAALSI). Int J Epidemiol 2018;47:689–90. 29325152 10.1093/ije/dyx247 PMC6005147

[29] Statistics South Africa. Statistical release Mid-year population estimates 2015, 2018.

[30] Kahn K, Collinson MA, Gómez-Olivé FX, Profile: Agincourt health and socio-demographic surveillance system. Int J Epidemiol 2012;41:988–1001. 22933647 10.1093/ije/dys115 PMC3429877

[31] Documentation of cognitive functioning measures in the health and retirement study | health and retirement study. Available: https://hrs.isr.umich.edu/publications/biblio/5620 [Accessed 29 Jul 2021].

[32] Kobayashi LC, Gross AL, Gibbons LE, You say tomato, I say radish: can brief cognitive assessments in the US health retirement study be harmonized with its international partner studies? J Gerontol B Psychol Sci Soc Sci 2020. doi:10.1093/geronb/gbaa205. [Epub ahead of print: 29 Nov 2020]. PMC8557836 33249448

[33] Bäckman L, Small BJ, Fratiglioni L. Stability of the preclinical episodic memory deficit in Alzheimer’s disease. Brain 2001;124:96–102. 11133790 10.1093/brain/124.1.96

[34] Kobayashi LC, Glymour MM, Kahn K, Childhood deprivation and later-life cognitive function in a population-based study of older rural South Africans. Soc Sci Med 2017;190:20–8. 28837862 10.1016/j.socscimed.2017.08.009 PMC5915343

[35] Pearl J. Lord’s Paradox Revisited – (Oh Lord! Kumbaya!). J Causal Inference 2016;4.

[36] Richiardi L, Bellocco R, Zugna D. Mediation analysis in epidemiology: methods, interpretation and bias. Int J Epidemiol 2013;42:1511–9. 24019424 10.1093/ije/dyt127

[37] Weuve J, Proust-Lima C, Power MC, Guidelines for reporting methodological challenges and evaluating potential bias in dementia research. Alzheimers Dement 2015;11:1098–109. 26397878 10.1016/j.jalz.2015.06.1885 PMC4655106

[38] Stern Y. What is cognitive reserve? theory and research application of the reserve concept. J Int Neuropsychol Soc 2002;8:448–60. 11939702

[39] Steffens DC, Otey E, Alexopoulos GS, Perspectives on depression, mild cognitive impairment, and cognitive decline. Arch Gen Psychiatry 2006;63:130. 16461855 10.1001/archpsyc.63.2.130

[40] Baumgart M, Snyder HM, Carrillo MC, Summary of the evidence on modifiable risk factors for cognitive decline and dementia: a population-based perspective. Alzheimer’s & Dementia 2015;11:718–26. doi:10.1016/j.jalz.2015.05.016 26045020

