# Supplementary material for: Subjective social position and cognitive function in a longitudinal cohort of older, rural South African adults, 2014–2019
Source: J Epidemiol Community Health. Author manuscript; Available in PMC 2023 Apr 1. (PMC8919590; doi:10.1136/jech-2021-217059)
Supplement: Supplementary file 1 [file NIHMS1764191-supplement-Supplementary_Material.pdf]

BMJ Publishing Group Limited (BMJ) disclaims all liability and responsibility arising from any reliance placed on this supplemental material which has been supplied by the author(s)

Subjective social position and cognitive function in a longitudinal cohort of older, rural South African adults, 2014-19

Supplemental Material

Contents:

Supplemental Methods 1. Details of the ISCO-08 (page 2)

Supplemental Methods 2. Inverse probability weights for mortality and attrition (page 5)

Supplementary Table 1. Logistic regression predicting survival at Wave 2 from Wave 1 characteristics**,** "Health and Aging in Africa: A Longitudinal Study of an INDEPTH Community in South Africa" (HAALSI), Agincourt sub-district, Mpumalanga, South Africa, 2014-19, N=5,059 (page 6)

Supplemental Table 2. Logistic regression predicting non-attrition due to refusal or not being found at Wave 2, conditional on survival from Wave 1 to Wave 2, "Health and Aging in Africa: A Longitudinal Study of an INDEPTH Community in South Africa" (HAALSI), Agincourt sub-district, Mpumalanga, South Africa, 2014-19, N=4,464 (page 7)

Supplemental Table 3. Comparison of sociodemographic characteristics of participants aged ≥40 years in the "Health and Aging in Africa: A Longitudinal Study of an INDEPTH Community in South Africa" (HAALSI) and the "National Income Dynamics Study" (NIDS) Wave 4, South Africa, 2014-15 (page 8)

Supplemental Table 4. Full output from linear regression models predicting baseline cognitive function score, "Health and Aging in Africa: A Longitudinal Study of an INDEPTH Community in South Africa" (HAALSI), Agincourt sub-district, Mpumalanga, South Africa, 2014-19, N=3,771 (page 9)

Supplemental Table 5. Full output from linear regression models predicting follow-up cognitive function score, "Health and Aging in Africa: A Longitudinal Study of an INDEPTH Community in South Africa" (HAALSI), Agincourt sub-district, Mpumalanga, South Africa, 2014-19, N=3,771 (page 10)

Kobayashi LC, *et al. J Epidemiol Community Health* 2021;0:1–6. doi: 10.1136/jech-2021-217059

Subjective social position and cognitive function in a longitudinal cohort of older, rural South African adults, 2014-19

Supplemental Methods 1: Details of the ISCO-08
The International Standard Classification of Occupation 2008 (ISCO-08) defines 9 major occupation groups (ISCO-08 major groups; not including armed forces, which is not applicable in the present study) which are classified into four skill levels: Low Skill (Level 1), Medium Skill (Level 2), and High Skill (Levels 3 and 4). The skill levels are defined by the ISCO-08 as follows:

Skill Level 1: Occupations at Skill Level 1 typically involve the performance of simple and routine physical or manual tasks. They may require the use of hand-held tools, such as shovels, or of simple electrical equipment, such as vacuum cleaners. They involve tasks such as cleaning; digging; lifting and carrying materials by hand; sorting, storing, or assembling goods by hand (sometimes in the context of mechanized operations); operating non-motorized vehicles; and picking fruit and vegetables. Many occupations at Skill Level 1 require physical strength and/or endurance. For some jobs basic skills in literacy and numeracy may be required. If required these skills would not be a major part of the work.

Skill Level 2: Occupations at Skill Level 2 typically involve the performance of tasks such as operating machinery and electronic equipment; driving vehicles; maintenance and repair of electrical and mechanical equipment; and manipulation, ordering, and storage of information. For almost all occupations at Skill Level 2 the ability to read information such as safety instructions, to make written records of work completed, and to accurately perform simple arithmetical calculations is essential. Many occupations at this skill level require relatively advanced literacy and numeracy skills and good interpersonal communication skills. In some occupations these skills are required for a major part of the work. Many occupations at this skill level require a high level of manual dexterity.

Skill Level 3: Occupations at Skill Level 3 typically involve the performance of complex technical and practical tasks that require and extensive body of factual, technical, and procedural knowledge in a specialized field. Examples of specific tasks performed include: ensuring compliance with health, safety, and related regulations; preparing detailed estimates of quantities and costs of materials and labor required for specific projects; coordinating, supervising, controlling, and scheduling the activities of other workers; and performing technical functions in support of professionals. Occupations at this skill level generally require a high level of literacy and numeracy and well-developed interpersonal communication skills. These skills may include the ability to understand complex written material, prepare factual reports and communicate verbally in difficult circumstances.

Skill Level 4: Occupations at Skill Level 4 typically involve the performance of tasks that require complex problem-solving, decision-making and creativity based on an extensive body of theoretical and factual knowledge in a specialized field. The tasks performed typically include analysis and research to extend the body of human knowledge in a particular field, diagnosis and treatment of disease, imparting knowledge to others, and design of structures or machinery and of processes for construction and production. Occupations at this skill level generally require extended levels of literacy and numeracy, sometimes at a very high level, and excellent interpersonal communication skills. These skills usually include the ability to understand complex written material and communicate complex ideas in media such as books, images, performances, reports, and oral presentations.

Further details on the ISCO-08 classifications and examples of job titles within each classification are available at: https://www.ilo.org/public/english/bureau/stat/isco/isco08/

Subjective social position and cognitive function in a longitudinal cohort of older, rural South African adults, 2014-19

According to the ISCO-08, we classified the 30 different types of occupations reported for the respondent's father's occupation into the ISCO-08 major occupation groups, and then into the four skill levels, as follows:

Raw variable value	Raw variable label in HAALSI	ISCO-08 Skill Level	ISCO-08 Major Group Number
1	Farm work	Low (1)	9 - Elementary occupations
2	Domestic work	Low (1)	9 - Elementary occupations
3	Construction work	Low (1)	9 - Elementary occupations
4	Security work	Medium (2)	5 - Services and sales workers
5	Cleaning work	Low (1)	9 - Elementary occupations
6	Small business owner	High (4)	1 - Managers
7	Mine work	Low (1)	9 - Elementary occupations
8	Teacher	High (4)	2 - Professionals
9	Traditional healer	High (3)	3 - Technicians and associate professionals
10	Health sector (formal)	High (4)	2 - Professionals
11	Game farm/game reserve (e.g. ranger)	Medium (2)	5 - Services and sales workers
12	Driver	Medium (2)	5 - Services and sales workers
13	Skilled worker (e.g. plumber, mechanic, electrician)	Medium (2)	7 - Craft and related trades workers
14	Cook/chef/catering	Medium (2)	5 - Services and sales workers
15	Unskilled worker (e.g. general labourer)	Low (1)	9 - Elementary occupations
16	Artisan (e.g. carpenter, wood carver, weaver)	Medium (2)	7 - Craft and related trades workers
17	Waiter/barman	Medium (2)	5 - Services and sales workers
18	Informal selling	Medium (2)	5 - Services and sales workers
19	Small business assistant	High (3)	3 - Technicians and associate professionals
20	Clerical and office work	Medium (2)	4 - Clerical support workers
21	Cattle herder	Low (1)	9 - Elementary occupations
22	Sewing, hairdressing, baking, brewing	Medium (2)	5 - Services and sales workers
23	Police, soldier, fireman	Medium (2)	5 - Services and sales workers
24	Petrol attendant	Medium (2)	5 - Services and sales workers
25	Timber, sawmill, poles	Medium (2)	8 - Plant and machine operators and assemblers
26	Gardening services	Low (1)	9 - Elementary occupations
27	Fieldworker - NGO or university	High (1)	2 - Professionals
28	Art, craft, photography, fashion design	High (1)	2 - Professionals

Subjective social position and cognitive function in a longitudinal cohort of older, rural South African adults, 2014-19

29	Senior administrator, manager, professional	High (1)	1 - Managers / 2 - Professionals
30	Priest/pastor	High (1)	2 - Professionals
31	Other	N/A	N/A
32	Unknown	N/A	N/A

Subjective social position and cognitive function in a longitudinal cohort of older, rural South African adults, 2014-19

Supplemental Methods 2: Inverse probability weights for mortality and attrition

We created inverse probability weights that jointly accounted for potential selection bias due to nonresponse to the wave 2 HAALSI interview for two key reasons: 1) mortality between waves 1 and 2, and 2) attrition due to reasons other than mortality between waves 1 and 2 (e.g., refusals to be interviewed or not found for contact).

We first used logistic regression to predict survival between waves 1 and 2 based on the following covariates measured at wave 1: age (continuous), sex (male; female), country of birth (South Africa; Mozambique or other), years of education (continuous), literacy (can read and write; cannot read or write), marital status (never married, currently married or living with a partner, separated/deserted, divorced, or widowed), employment status (employed full- or part-time; not working; homemaker), household per capita consumption quintiles, cognitive function score (composite of orientation, immediate word recall, delayed word recall, and two numeracy items), CES-D depression scale score (continuous), grip strength (continuous; the maximum grip strength value recorded over four measurement sessions), average 2.5 meter walk time (continuous), HIV status based on dried blood spot measures (positive; negative or indeterminate/missing), whether respondent was missing HIV data (yes; no), HIV viral load (0; <100; 100-400; 400-1000; 1000-10,000; >10,000 copies/mL), and whether the respondent had a proxy interview (yes; no). Mean or mode imputation was used to impute missing values for a small number of individuals with missing data on certain variables. The full logistic regression model that was used to predict survival is shown in Supplementary Table 1.

We then used logistic regression to predict non-attrition between waves 1 and 2 based on the following covariates measured at wave 1: age (categorical), sex (male; female), country of birth (South Africa; Mozambique or other), years of education (continuous), literacy (can read and write; cannot read or write), marital status (never married, currently married or living with a partner, separated/deserted, divorced, or widowed), employment status (employed full- or part-time; not working; homemaker), household per capita consumption quintiles, cognitive function score (composite of orientation, immediate word recall, delayed word recall, and two numeracy items), whether the respondent had a proxy interview (yes; no), migration status (measured at wave 2, capturing whether the respondent had moved out of Agincourt but remained in Mpumalanga, migrated to another province in South Africa, or migrated to another country), participation in other local research studies (yes; no), month of first contact for the wave 2 interview, and time of day of first contact for the wave 2 interview. Mean or mode imputation was used to impute missing values for a small number of individuals with missing data on certain variables. The full logistic regression model that was used to predict non-attrition is shown in Supplementary Table 2.

The final inverse probability weight was calculated by taking the inverse of each of the survival and non-attrition probabilities estimated by the two logistic regression models and multiplying them together. We considered truncating the weight at the 99th percentile, but we did not do so because there were no extreme outlying individual weights. This final joint mortality and attrition weight was applied to all linear and quantile models in the present study.

Subjective social position and cognitive function in a longitudinal cohort of older, rural South African adults, 2014-19

Supplemental Table 1. Logistic regression predicting survival at Wave 2 from Wave 1 characteristics**,** "Health and Aging in Africa: A Longitudinal Study of an INDEPTH Community in South Africa" (HAALSI), Agincourt sub-district, Mpumalanga, South Africa, 2014-19, N=5,059

Characteristic	Odds Ratio	95% CI
Female sex	2.544	(2.031, 3.187)
Age (per year)	0.954	(0.946, 0.963)
Born in South Africa	0.797	(0.644, 0.987)
Years of education	1.006	(0.971, 1.043)
Literacy	0.940	(0.724, 1.222)
Marital status		
Married or living with partner	-	(reference)
Never married	0.516	(0.338, 0.787)
Separated or deserted	0.747	(0.516, 1.081)
Divorced	0.494	(0.327, 0.747)
Widowed	0.723	(0.562, 0.929)
Employment status		
Not working	-	(reference)
Employed	1.378	(0.943, 2.016)
Homemaker	1.025	(0.748, 1.403)
Household consumption quintile		
1 (lowest consumption)	-	(reference)
2	1.115	(0.839, 1.482)
3	1.207	(0.904, 1.612)
4	1.223	(0.913, 1.637)
5 (highest consumption)	1.119	(0.825, 1.517)
Total cognitive score	1.041	(1.016, 1.066)
CESD-8 depression scale	0.908	(0.860, 0.959)
Grip strength	1.025	(1.014, 1.036)
Average walk time	0.986	(0.961, 1.011)
HIV positive		
HIV negative	-	(reference)
HIV positive	1.061	(0.701, 1.608)
Missing on HIV	0.677	(0.507, 0.904)
HIV viral load	0.785	(0.694, 0.888)
Proxy interview	0.306	(0.189, 0.495)
_cons	70.279	(29.479, 167.549)

Note: c-statistic was 0.7678

Subjective social position and cognitive function in a longitudinal cohort of older, rural South African adults, 2014-19

Supplemental Table 2. Logistic regression predicting non-attrition due to refusal or not being found at Wave 2, conditional on survival from Wave 1 to Wave 2, "Health and Aging in Africa: A Longitudinal Study of an INDEPTH Community in South Africa" (HAALSI), Agincourt sub-district, Mpumalanga, South Africa, 2014-19, N=4,464

Characteristic	Odds Ratio	95% CI
Female sex	1.060	(0.810, 1.389)
Age group		
40-49	-	(reference)
50-59	0.708	(0.491, 1.021)
60-69	0.688	(0.433, 1.093)
70-79	0.935	(0.537, 1.629)
80+	0.903	(0.448, 1.819)
Born in South Africa	0.764	(0.550, 1.060)
Years of education	0.949	(0.913, 0.986)
Literacy	0.990	(0.699, 1.401)
Marital status		
Married or living with partner	-	(reference)
Never married	0.958	(0.583, 1.575)
Separated or deserted	1.469	(0.894, 2.416)
Divorced	0.891	(0.514, 1.545)
Widowed	1.386	(0.973, 1.973)
Employment status		
Not working	-	(reference)
Employed	0.720	(0.523, 0.991)
Homemaker	1.149	(0.729, 1.813)
Household consumption		
1 (lowest consumption)	-	(reference)
2	1.040	(0.705, 1.532)
3	1.040	(0.705, 1.535)
4	1.095	(0.733, 1.635)
5 (highest consumption)	1.405	(0.931, 2.122)
Total cognitive score	0.982	(0.951, 1.013)
Proxy interview	0.564	(0.184, 1.729)
Migration status at wave 2	0.200	(0.142, 0.283)
Participation in other local studies	0.884	(0.634, 1.232)
Month of first contact at wave 2		
January 2018	1.789	(1.258, 2.543)
February 2018	1.147	(0.731, 1.799)
March-April 2018	0.647	(0.237, 1.765)
July-October 2018	1.758	(1.234, 2.507)
November 2018	-	(reference)
December 2018	1.038	(0.716, 1.504)
Time of day of first contact at wave 2		
Morning	-	(reference)
Afternoon/Evening	0.749	(0.563, 0.996)
_cons	32.582	(16.625, 63.856)

Note: c-statistic was 0.6987

Subjective social position and cognitive function in a longitudinal cohort of older, rural South African adults, 2014-19

Supplemental Table 3. Comparison of sociodemographic characteristics of participants aged ≥40 years in the "Health and Aging in Africa: A Longitudinal Study of an INDEPTH Community in South Africa" (HAALSI) and the "National Income Dynamics Study" (NIDS) Wave 4, South Africa, 2014-15

Characteristic	HAALSI Unweighted	NIDS Unweighted	NIDS Weighted[a]
	3,117 (100%)	10,827 (100%)	Population size: 16,909,756
Age			
Mean (SD, Range)	60.1 (12.2, 40-111)	56.1 (12.0, 40-113)	54.9 (54.5, 55.34)
Sex			
Male	1,657 (43%)	4,199 (39%)	44% (42%, 46%)
Female	2,114 (56%)	6,628 (61%)	56% (54%, 58%)
Race/population group			
African	3,117 (100%)	8,019 (74%)	73% (71%, 75%)
Coloured	0 (0%)	1,744 (16%)	10% (9%, 11%)
Asian/Indian	0 (0%)	197 (2%)	3% (2%, 4%)
White	0 (0%)	867 (8%)	14% (13%, 16%)
Education			
No formal education	1,605 (43%)	2,061 (19%)	13% (12%, 14%)
Some primary (1-7 years)	1,343 (36%)	3,175 (29%)	27% (25%, 28%)
Some secondary (8-11 years)	464 (12%)	2,455 (23%)	27% (26%, 29%)
Secondary or more (≥12 years)	359 (10%)	948 (9%)	32% (31%, 34%)
Unknown or other	0 (0%)	948 (9%)	<1%
Marital status			
Married or living as married	2,024 (54%)	4,740 (51%)	57% (55%, 60%)
Never married	181 (5%)	2,062 (22%)	18% (17%, 19%)
Divorced/separated/deserted	477 (12%)	414 (5%)	6% (5%, 7%)
Widowed	1,089 (29%)	2,154 (23%)	19% (18%, 20%)
Employment status[b]			
Employed part or full-time	657 (17%)	3,788 (41%)	50% (48%, 52%)
Not working	2,695 (71%)	N/A	N/A
Homemaker	419 (11%)	N/A	N/A
Not economically active	N/A	4,740 (51%)	43% (41%, 45%)
Unemployed	N/A	653 (7%)	7% (6%, 8%)
Unknown	0 (0%)	24 (<1%)	<1%

Note: HAALSI is representative of the Agincourt sub-district, Mpumalanga province, South Africa, and NIDS is nationally representative of South Africa. The data presented in this table were collected in 2014-15 for both studies, and represent the populations aged ≥40 years in both samples. Column totals for some variables for the unweighted NIDS sample may not sum to 10,827 due to missing data.
[a]The weighted distributions are presented as percentages and 95% confidence intervals around the percentages. The weighted estimates were generated using the NIDs wave 4 panel weights, which correct for non-response to the original wave 1 survey, panel attrition between waves 1 and 4, and are calibrated to provincial population totals and to gender-age group-race cell totals.
[b]Categories for the current employment status variable differed between HAALSI and NIDS. Variable categories that did not apply within each study are indicated as "N/A" cells in the table.

Subjective social position and cognitive function in a longitudinal cohort of older, rural South African adults, 2014-19

Supplemental Table 4. Full output from linear regression models predicting baseline cognitive function score, “Health and Aging in Africa: A Longitudinal Study of an INDEPTH Community in South Africa” (HAALSI), Agincourt sub-district, Mpumalanga, South Africa, 2014-19, N=3,771

Covariate	Mean change in baseline cognitive function score (range: 0-24)					
	Model 1[a]		Model 2[c]		Model 3[c]	
	β	95% CI	β	95% CI	β	95% CI
Intercept	16.915	(13.980, 19.851)	9.399	(6.652, 12.147)	8.858	(6.116, 11.600)
SSP (per ladder rung increase)	0.300	(0.244, 0.356)	0.210	(0.156, 0.264)	0.198	(0.144, 0.253)
Age (per year)	-0.086	(-0.182, 0.010)	0.049	(-0.036, 0.135)	0.0587	(-0.027, 0.145)
Age^2	-0.000	(-0.001, 0.000)	-0.001	(-0.002, -0.000)	-0.001	(-0.002, -0.000)
Sex (female vs. male)	-0.663	(-0.916, -0.411)	-0.003	(-0.270, 0.263)	-0.079	(-0.347, 0.189)
Country of birth						
South Africa	-	(ref)	-	(ref)	-	(ref)
Mozambique or other	-1.133	(-1.409, -0.858)	0.051	(-0.250, 0.352)	0.068	(-0.228, 0.363)
Father’s occupation						
Unskilled manual labor			-	(ref)	-	(ref)
Mining or service industry			0.554	(0.273, 0.836)	0.561	(0.285, 0.837)
Traditional healer or assistant			-0.192	(-0.968, 0.584)	-0.004	(-0.774, 0.766)
Professional or managerial			0.702	(-0.047, 1.45)	0.710	(-0.045, 1.465)
Other			0.220	(-0.171, 0.611)	0.190	(-0.190, 0.571)
Don’t know			-0.370	(-0.811, 0.070)	-0.340	(-0.779, 0.099)
Education						
No formal education			-	(ref)	-	(ref)
Some primary (1-7 years)			0.622	(0.262, 0.982)	0.660	(0.310, 1.011)
Some secondary (8-11 years)			1.062	(0.565, 1.556)	1.086	(0.607, 1.565)
Secondary or more (12+ years)			2.112	(1.514, 2.710)	2.224	(1.639, 2.809)
Self-reported literacy						
Cannot read or write			-	(ref)	-	(ref)
Can read or write			1.922	(1.585, 2.259)	1.820	(1.491, 2.150)
Marital status						
Married or living as married			-	(ref)	-	(ref)
Never married			0.094	(-0.567, 0.754)	0.060	(-0.586, 0.705)
Separated or deserted			-0.570	(-1.015, -0.124)	-0.538	(-0.971, -0.106)
Divorced			-0.107	(-0.706, 0.493)	-0.068	(-0.681, 0.545)
Widowed			-0.562	(-0.867, -0.258)	-0.559	(-0.860, -0.258)
Employment status						
Employed (part- or full-time)			-	(ref)	-	(ref)
Not working			-0.249	(-0.592, 0.0946)	-0.146	(-0.479, 0.187)
Homemaker			0.163	(-0.291, 0.617)	0.208	(-0.241, 0.656)
Household asset quintile						
1 (poorest)			-	(ref)	-	(ref)
2			0.013	(-0.379, 0.409)	0.011	(-0.370, 0.391)
3			0.170	(-0.221, 0.561)	0.168	(-0.216, 0.553)
4			0.172	(-0.219, 0.563)	0.132	(-0.248, 0.512)
5 (richest)			0.505	(0.092, 0.918)	0.467	(0.067, 0.867)
Self-rated health today vs. 1 year ago						
Much worse					0.375	(-0.392, 1.142)
Worse					-0.054	(-0.379, 0.270)
Same					-	(ref)
Better					0.658	(0.331, 0.985)
Much better					2.854	(2.257, 3.450)
Depressive symptoms (per symptom)					-0.043	(-0.360, 0.273)
Frequency of alcohol consumption						
<5 days per week					-	(ref)
≥5 days per week					-0.699	(-1.264, -0.133)
Diabetes (yes vs. no)					-0.128	(-0.349, 0.093)
Hypertension (yes vs. no)					0.087	(-0.155, 0.329)

Note: All models incorporate IPWs for mortality and attrition
[a]Adjusted for Model 1 covariates, plus socioeconomic and social factors (father’s occupation, education, literacy, marital status, employment status, household asset quintile)

Kobayashi LC, *et al. J Epidemiol Community Health* 2021;0:1–6. doi: 10.1136/jech-2021-217059

Subjective social position and cognitive function in a longitudinal cohort of older, rural South African adults, 2014-19

[b]Adjusted for Model 1 and 2 covariates, plus health-related factors (self-rated health today compared to one year ago, alcohol intake frequency, number of depressive symptoms, diabetes, hypertension)

Supplemental Table 5. Full output from linear regression models predicting follow-up cognitive function score, "Health and Aging in Africa: A Longitudinal Study of an INDEPTH Community in South Africa" (HAALSI), Agincourt sub-district, Mpumalanga, South Africa, 2014-19, N=3,771

Covariate	Mean change in baseline cognitive function score (range: 0-24)					
	Model 1[a]		Model 2[c]		Model 3[c]	
	β	95% CI	β	95% CI	β	95% CI
Intercept	17.634	(13.897, 21.370)	11.203	(7.777, 14.629)	11.238	(7.812, 14.664)
SSP (per ladder rung increase)	0.168	(0.110, 0.226)	0.081	(0.024, 0.138)	0.078	(0.021, 0.136)
Age (per year)	-0.004	(-0.127, 0.121)	0.109	(-0.002, 0.220)	0.111	(0.000, 0.223)
Age^2	-0.001	(-0.002, -0.000)	-0.002	(-0.003, -0.001)	-0.002	(-0.003, -0.001)
Sex (female vs. male)	-1.094	(-1.350, 0.226	-0.614	(-0.891, -0.337)	-0.684	(-0.967, -0.401)
Country of birth						
South Africa	-	(ref)	-	(ref)	-	(ref)
Mozambique or other	1.644	(-1.933, 1.35)	-0.514	(-0.838, -0.191)	-0.503	(-0.827, -0.179)
Father's occupation						
Unskilled manual labor			-	(ref)	-	(ref)
Mining or service industry			0.398	(0.108, 0.688)	0.397	(0.107, 0.688)
Traditional healer or assistant			-0.317	(-1.07, 0.438)	-0.272	(-1.034, 0.491)
Professional or managerial			0.154	(-0.525, 0.833)	0.152	(-0.528, 0.832)
Other			0.302	(-0.129, 0.734)	0.314	(-0.119, 0.748)
Don't know			0.071	(-0.374, 0.516)	0.078	(-0.365, 0.521)
Education						
No formal education			-	(ref)	-	(ref)
Some primary (1-7 years)			0.788	(0.418, 1.158)	0.786	(0.416, 1.56)
Some secondary (8-11 years)			1.739	(1.251, 2.226)	1.723	(1.234, 2.211)
Secondary or more (12+ years)			2.133	(1.597, 2.667)	2.131	(1.593, 2.668)
Self-reported literacy						
Cannot read or write			-	(ref)	-	(ref)
Can read or write			1.246	(0.901, 1.592)	1.243	(0.898, 1.588)
Marital status						
Married or living as married			-	(ref)	-	(ref)
Never married			-0.845	(-1.432, -0.258)	-0.795	(-1.386, -0.204)
Separated or deserted			-0.524	(-0.978, -0.069)	-0.525	(-0.978, -0.204)
Divorced			-0.619	(-1.262, 0.023)	-0.604	(-1.245, 0.380)
Widowed			-0.303	(-0.624, 0.019)	-0.302	(-0.625, 0.022)
Employment status						
Employed (part- or full-time)			-	(ref)	-	(ref)
Not working			-0.247	(-0.573, 0.078)	-0.221	(-0.547, 0.106)
Homemaker			-0.292	(-0.760, 0.174)	-0.322	(-0.790, 0.146)
Household asset quintile						
1 (poorest)			-	(ref)	-	(ref)
2			0.260	(-0.148, 0.667)	0.239	(-0.168, 0.646)
3			0.663	(0.248, 1.078)	0.623	(0.208, 1.038)
4			0.623	(0.200, 1.046)	0.586	(0.162, 1.010)
5 (richest)			0.967	(0.531, 1.402)	0.924	(0.487, 1.361)
Self-rated health today vs. 1 year ago						
Much worse					-0.137	(-0.911, 0.637)
Worse					-0.352	(-0.715, 0.011)
Same					-	(ref)
Better					-0.081	(-0.412, 0.250)
Much better					-0.051	(-0.564, 0.462)
Depressive symptoms (per symptom)					0.120	(-0.224, 0.463)
Frequency of alcohol consumption						
<5 days per week					-	(ref)
≥5 days per week					-0.591	(-1.141, -0.041)
Diabetes (yes vs. no)					-0.147	(-0.356, 0.063)
Hypertension (yes vs. no)					0.162	(-0.090, 0.415)

Subjective social position and cognitive function in a longitudinal cohort of older, rural South African adults, 2014-19

Note: All models incorporate IPWs for mortality and attrition
[a]Adjusted for Model 1 covariates, plus socioeconomic and social factors (father's occupation, education, literacy, marital status, employment status, household asset quintile)
[b]Adjusted for Model 1 and 2 covariates, plus health-related factors (self-rated health today compared to one year ago, alcohol intake frequency, number of depressive symptoms, diabetes, hypertension)

Kobayashi LC, *et al. J Epidemiol Community Health* 2021;0:1–6. doi: 10.1136/jech-2021-217059
